# Bias in Auditory Perception

**DOI:** 10.1177/2041669515607153

**Published:** 2015-10-13

**Authors:** Marjoleine Sloos, Denis McKeown

**Affiliations:** Interacting Minds Centre, Aarhus University, Aarhus, Denmark; School of Psychology, University of Leeds, UK

Our brain accomplishes the remarkable feat of processing a continuous stream of incoming sensory information at an astonishing speed. This is possible through simultaneous bottom-up processing of the incoming stimuli and top-down processing of prior knowledge (Kinchla & Wolfe, 1979). Categorization facilitates recognition and also anticipation of the incoming stimuli (also known as predictive coding, e.g., Rao & Ballard, 1999). Therefore, the presentation of written words facilitates the auditory perception of distorted pronunciation (Sohoglu, Peelle, Carlyon, & Davis, 2012). The skill to correctly categorize depends on relevant experience and memory that has been built over time. Perception thus involves a balance between the sensory stimuli and the stored representations in memory. If a category or a mental image is inaccurately matched with the incoming stimuli, biased perception emerges (see examples later). Bias in perception occurs mostly unconsciously and perhaps incessantly—after all, stimuli are unlikely to form a perfect match with stored memory.

Perceptual bias is related to cognitive bias, like gender bias, (unintended) discrimination, and placebo effects. The mechanism is the same, viz. the interference of previously stored impressions, information, and knowledge with newly incoming (perceptual or cognitive) information. Most research on bias takes either a cross-modal or a domain-general (rather than a domain-specific) perspective. A textbook example of cross-modal bias in language is the McGurk effect, in which the *auditory* presentation of a speech sound [IPA ga] and the simultaneous *visual* presentation of another sound [ba] lead to a blended perception resulting in [da] (McGurk & MacDonald, 1976). Cross-modal perception has been reported for all sensory modes. For instance, biased gustatory perception may arise as a result of food color (Spence, Levitan, Shankar, & Zampini, 2010). Also, vision is influenced by tactile perception and vice versa, for example, in an experiment in which subjects were presented with simultaneous tactile taps and visual flashes (Bresciani et al., 2005). Domain-general bias occurs when cognitive representations bias the interpretation of sensory stimuli. For example, information about the health consequences of acetone smell biases the reports on how the subjects felt after exposure to the smell of acetone (Dalton, Wysocki, Brody, & Lawley, 1997).

This leads us to the direction of bias, which is based on expectations. If memory is activated when processing perceptual stimuli, expectations may arise about the exact nature of those stimuli based on prior knowledge. That is the reason why someone who is informed that the odor of acetone is bad for one’s health is more likely to report he feels not well after exposure to the smell of acetone. Similarly, Spence et al. (2010) argue that it is the expectation that food color raises, which leads to misidentification or misperception of the taste. Expectations play an important role in auditory perception as well, for instance, in language.

Research on (modal-specific) auditory bias and the effect of expectations in language seems limited at first glance, compared with research on cognitive and cross-modal biases. However, this is a matter of terminology, and a lack of recognition that different subdisciplines within linguistics actually make common observations, and similarly between language and music cognition, as well as between psychology and linguistics. The concept of biased perception in language was described as early as 1889 in a case of self-observation of the field linguist Boas (1889).^11^ Numerous studies since then have shown that second language perception is influenced by the phonemic representations of the native language, and models have been developed to account for L2 perception in relation to the L1 phonemic inventories (Best & Tyler, 2007; Flege, 1992; Kuhl, 1992). Another field in which biased perception has repeatedly been observed is sociolinguistics. Speakers differ in pronunciation based on, among other things, their gender, age, the social class they identify themselves with, and dialectal accent. Listeners habitually categorize the speaker into a certain age-group or social or economic group. But miscategorization easily leads to biased perception. Listeners, who are misinformed about a speaker’s (socio-)linguistic background, are more inclined to perceive the incoming stimulus according to their sociolinguistic expectations than to the acoustic characteristics of the stimulus (see Drager, 2010 for an extensive overview). Apart from misinformation about the speaker, it may just be difficult to categorize a particular speech variety, due to a lack of experience. In such cases, listeners tend to perceive a speaker as sounding similar to themselves (Koops, 2011).

This special issue contains a collection of selected articles which were presented at the international conference “Bias in Auditory Perception” held in Aarhus, September 18–20, 2014. The conference covered a broad array of topics in which biased auditory perception has been observed. Linguists, neuroscientists, musicians, and psychologists from all over the world found common ground in recognizing patterns of interference of cognition, other perceptual stimuli, or mood, with the incoming speech sound, musical string, or even the cry of an infant. The subjects contained in this special issue are also broad, although they all fall within the realm of linguistics. Not only domain-specific bias is discussed in this issue. As van der Ham and de Boer show, domain-general bias can play a role in perception of speech sounds as well: Linguistically speaking, the presentation of a continuum of two contrastive speech sounds is expected to lead to binary parsing, but without communicative function, these sounds are perceived as single category. Biased perception in second language acquisition is reported in Wang and van Heuven, who address the topic of mutual intelligibility, showing that speakers of a second language are better understood by listeners who share the same native language than by native speakers of the target language. Katayama compares the perception of English syllable structure by native speakers and Japanese learners of English at two proficiency levels, finding the three groups adopt different strategies. Nielsen et al. show that first language bias can be overcome within three weeks of intensive training in a foreign language. Further, Gilbers et al. show that listeners with cochlear implants may have other biases than listeners with normal hearing. Whereas the first group is biased toward pitch range as the most important cue for emotional intonation, the latter is biased toward pitch contour as the most salient cue. Finally, Sloos and Ariza observe an own-variety bias in which native speakers who are invited to participate in a language variety identification task report to perceive their own variety even if it is not presented at all. We hope that these contributions raise the awareness of the common role of expectancies and observations of bias in different subdisciplines of linguistics and that it will form the starting point for a more interdisciplinary approach to perceptual bias, in which auditory bias is related to other perceptual modes and cognition.

## Note

1 As pointed out in the keynote talk “In the mind of the listener: Enculturing through patterned practice”, by Andreas Roepstorff at the “Bias in Auditory Perception” conference.
